# Germination and Growth of Spinach under Potassium Deficiency and Irrigation with High-Salinity Water

**DOI:** 10.3390/plants9121739

**Published:** 2020-12-09

**Authors:** Kadir Uçgun, Jorge F. S. Ferreira, Xuan Liu, Jaime Barros da Silva Filho, Donald L. Suarez, Claudivan F. de Lacerda, Devinder Sandhu

**Affiliations:** 1 Department of Plant and Animal Production, Technical Sciences Vocational School, Karamanoğlu Mehmetbey University, Karaman 70200, Turkey; kadirucgun@kmu.edu.tr; 2 US Salinity Laboratory (USDA-ARS), 450 W. Big Springs Rd., Riverside, CA 92507, USA; xuan.liu@usda.gov (X.L.); donald.suarez@usda.gov (D.L.S.); devinder.sandhu@usda.gov (D.S.); 3 Departments of Microbiology and Plant Pathology, University of California Riverside, 900 University Ave., Riverside, CA 92521, USA; jaimeba@ucr.edu; 4 Department of Agricultural Engineering, Federal University of Ceará, Fortaleza-CE 60450-760, Brazil; cfeitosa@ufc.br

**Keywords:** *Spinacia oleracea*, potassium deficiency, high-salinity water, potassium–salinity interaction, salt-tolerant glycophyte

## Abstract

Information is scarce on the interaction of mineral deficiency and salinity. We evaluated two salt-tolerant spinach cultivars under potassium (K) doses (0.07, 0.15, 0.3, and 3.0 mmol_c_ L^−1^) and saline irrigation (5, 30, 60, 120, and 160 mmol_c_ L^−1^ NaCl) during germination and growth. There was no interaction between salinity and K. Salinity decreased germination percent (GP), not always significantly, and drastically reduced seedling biomass. ‘Raccoon’ significantly increased GP at 60 mmol_c_ L^−1^ while ‘Gazelle’ maintained GP up to 60 or 120 mmol_c_ L^−1^. After 50 days under saline irrigation, shoot biomass increased significantly at 30 and 60 mmol_c_ L^−1^ at the lowest K dose but, in general, neither salinity nor K dose affected shoot biomass, suggesting that salinity supported plant growth at the most K-deficient dose. Salinity did not affect shoot N, P, or K but significantly reduced Ca, Mg, and S, although plants had no symptoms of salt toxicity or mineral deficiency. Although spinach seedlings are more sensitive to salt stress, plants adjusted to salinity with time. Potassium requirement for spinach growth was less than the current crop recommendation, allowing its cultivation with waters of moderate to high salinity without considerable reduction in yield, appearance, or mineral composition.

## 1. Introduction

The undesirable effects of salt stress on plant growth can be associated with (1) soil solution with low osmotic potential (water stress), (2) nutrient imbalance caused by excessive NaCl or by high soil pH, (3) specific ion effect (Na^+^ and/or Cl^−^), or (4) a combination of all three factors [1]. These factors cause adverse effects on plant growth and yield [2,3] and, when severe and prolonged, will lead to plant death. The most prevalent ions in salt-affected soils or saline irrigation water are Na^+^ and Cl^−^ and the great majority of salt stress is due to either one or both ions because they are highly soluble and become toxic to plants at high concentrations. These ions compete with the absorption of K^+^, Ca^2+^, and NO3^−^ and often lead to pertinent mineral nutrient imbalance and deficiency [4]. Recent reports indicate that the effects of competition between different ions depend on their tissue concentration and may vary with plant species [5,6,7].

Depending on water or soil salinity, Na and Cl can accumulate in spinach shoots at levels as high as the macronutrients N and K [6,8], indicating the existence of an efficient vacuolar sequestration of potentially toxic Na^+^ and Cl^−^ ions in this species. On the contrary, many glycophytes, including strawberry and Jerusalem artichoke, have mechanisms that prevent Na^+^ toxicity by reducing Na^+^ transport to leaves [5,9]. However, these mechanisms are not so efficient in controlling Cl^−^ absorption and translocation in most glycophytic plants, making Cl^−^ one of the main ions responsible for specific-ion toxicity. Thus, Cl alone, or associated with Na, may decrease plant performance after their excessive tissue accumulation [5,10].

Germination is highly influenced by environmental factors [11] and is a complex phenomenon involving many physiological and biochemical changes that are easily compromised by stress, including salt stress [12]. Salinity can affect the germination of seeds either by lowering soil osmotic potential and reducing water uptake by plant roots or by toxic effects caused by specific ions [13]. Salinity stress can change the activity of enzymes of the nucleic acid metabolism [14], alter protein metabolism [15,16], disturb hormonal balance [17], and reduce the utilization of seed reserves [18,19].

Data from several crops suggest that the level of salinity tolerance is highly dependent on the plant species [20,21], the cultivar within a species [9,22,23,24] or on the developmental stage of the plant [25]. Several researchers investigated the effect of salinity on the germination of plants like beans [26], quinoa [27,28], tomato [29], cabbage [30], cauliflower [30], canola [30,31], cowpea [32], onion [33], safflower [34], sunflower [35], eggplant [36] carrot [37], and spinach [20]. Salinity reduced germination rates in all of them.

Although it is known that salinity affects plant developmental stages differently, it is not known whether this would be associated with the duration of each stage or with the existence of specific mechanisms to maintain needed tissue levels of mineral nutrients for plant growth. For example, it is known that excess NaCl affects the absorption and accumulation of K in the leaves of many species, resulting in damage to cellular metabolism. In salt-tolerant species, however, Na may partially replace K to a great extent, but without damaging plant growth [38]. Thus, an important question arises: is this partial replacement of K by Na maintained and/or beneficial at all stages of the crop cycle? Furthermore, although it is known that salinity may affect the absorption of major mineral ions such as K^+^ and NO_3_^−^ in most glycophytes, there is no published data reporting the effect of moderate to high salinity of irrigation water, combined with deficient soil concentrations of either NO_3_^−^ or K^+^, on spinach germination and growth. Even data on ideal mineral (N, P, and K) nutrition for spinach without salinity was lacking until recently [39].

Considering that spinach is a moderately salt-tolerant glycophyte [6,8,40], we hypothesized that irrigation waters providing elevated NaCl-induced salinity combined with potassium deficiency would mimic similar conditions found in soil solutions, thus providing a model system to study spinach response to these stresses at both germination and late growth stage. Thus, the objective of this research was to examine the salt tolerance of two cultivars of spinach at different developmental stages (germination, seedling establishment, and vegetative growth) under cultivation with elevated salinity combined with K deficiency applied through the irrigation water.

## 2. Materials and Methods

### 2.1. Greenhouse Cultivation Details

Riverside, California, is situated at an altitude of 311 m with a latitude of 33.9°57′54″ N and a longitude of 117.3°20′13″ W. Although ideal temperatures for spinach growth are between 15 and 18 °C, the crop grows well in temperatures ranging from 5 to 30 °C [41]. Thus, greenhouse temperature control was set between 15 °C (night) and 28 °C (day). The greenhouse cultivation of both experiments spun from 19 October 2018 to 11 March 2019. During that time, spinach plants were grown under short days with 13–13.5 h dark/10.5 to 11.0 h of natural light, which is under the flowering inductive photoperiod (14 h day^−1^) for spinach [42]. Minimum to maximum greenhouse average relative humidity, temperature, day length, and light intensity are shown in Table 1.

Two experiments were conducted to evaluate the effects of NaCl doses and K deficiency, separately and combined, under greenhouse conditions on two spinach (*Spinacia oleracea* L.) cultivars (Raccoon and Gazelle). One experiment focused on assessing germination and seedling establishment (4–6 true leaves). The other experiment was carried out to assess vegetative growth, when salinity was applied to plants with 6–8 true leaves and continued for seven weeks until harvest. The pots were arranged in a randomized complete block design with four replicates, each with 3 plants per pot. Both experiments had 20 treatments assigned to each cultivar (combination of 5 NaCl concentrations with 4 K doses: 3 deficient and 1 control) with a total of 80 pots per cultivar, in a factorial arrangement.

For Experiment 1 (germination and seedling establishment), five seeds were sown directly at a depth of 1.3 cm in 5.7-L pots (22.2 cm in diameter) on 07 February 2019. Pots were filled with non-washed, non-sterile, sand and saturated with the pertinent treatment solution. After sowing, pots were irrigated three times a week with each saline treatment solution. Data on seed gemination were recorded daily between 13 and 32 days after sowing for each cultivar. All Seedlings from germinated seeds (no thinning was performed in this experiment) were harvested on the 32nd day after sowing 11 March 2019) with shoots separated from roots and recorded for shoot fresh weight (SFW). The coefficient of velocity of germination (CVG in % day^−1^) and the germination percentage (GP) at 13, 22, and 32 days were obtained according to [43], as follow:

CVG (% day^−1^) = [∑Ni/∑(NiTi)] × 100, where N was the number of seeds germinated on day i, Ti is the number of days from sowing and GP (%) = No. of seeds germinated per cultivar at: 13 days for ‘Gazelle’, 22 days for ‘Raccoon’, and 32 days for both cultivars × 100.

For Experiment 2 (vegetative growth), 7 seeds were sown directly into pots at 1.3 cm depth. Seeds were sown on 19 October 2018 and harvested on 28 January 2019 (101 days). Pots had the same dimensions and growth medium as described above. After germination, plants were thinned to the most homogeneous three plants per pot. Plants were watered with 1/8-strength Hoagland’s nutrition solution [44] made with Riverside municipal water with an electrical conductivity (EC_iw_) of 0.7 dS m^−1^ three times a week until the growth stage of 6–8 true leaves. Salinity treatments were applied on 10 December 2018 and continued until 28 January 2019. Before treatments were applied, all pots were flushed four times with deionized water to remove any remaining fertilizer (including K) from the sand. After that, treatments combining Na (as NaCl) and K (substituting K for Na) began and continued for 7 weeks (50 days) with harvest taking place from 29 January to 1 February 2019. All solutions (control and salt treatments) contained all macronutrients and micronutrients needed for spinach growth, as previously determined [6]. Solution volumes used to water plants were determined to allow leaching of approximately 30% (water volume drained/volume applied) for both experiments. At harvest, all three plants in each pot were separated into shoots and roots. Unwashed shoots were weighed immediately upon harvest to obtain shoot fresh weight (SFW, g pot^−1^). Then, shoots and roots were washed with tap water, twice with deionized water to remove remaining sand (roots) and mineral fertilizers from tissue surface (shoots and roots) before drying in a forced-air oven (70 °C) for at least 48 h to obtain constant shoot and root dry weight (SDW and RDW, respectively) in g pot^−1^ before grinding for mineral analysis.

### 2.2. Plant Mineral Analyses

In experiment 1 (germination and seedling establishment) plants were too small for mineral analysis and only the fresh weight of shoots was recorded. For Experiment 2, plants were harvested, separated into shoots and roots, washed with tap water, followed by deionized water and blotted dry with paper towels and bagged to be dried in a forced-air oven at 70 °C until stable dry weight. Dry weight was recorded for shoots and roots. Samples were then ground in a Wiley mill to pass a 20-mesh (0.84 mm) screen. Tissue mineral concentration was based on shoot or root dry weight. Chloride was determined from nitric/acetic acid extracts by amperometric titration. The concentrations of tissue Na, P, K, Ca, Mg, and total-S, and of the micronutrients Fe, Cu, Mn, Zn, and Mo were determined from nitric acid digestions (Milestone, Ethos EZ Microwave Digestion, Shelton, CT, USA) of the dried, ground, plant material by Inductively Coupled Plasma Optical Emission Spectrometry (ICP-OES, 3300DV, Perkin-Elmer Corp., Waltham, MA, USA). Nitrogen was determined by combustion in a Rapid N Exceed® analyzer (rNex, Elementar Americas Inc., Ronkonkoma, New York, NY, USA).

### 2.3. Saline Solutions

Saline solutions of the same salt composition were used as treatments in both experiments. In all, there were 20 treatments containing 5 nominal concentrations of Na (5, 30, 60, 120, and 160 mmol L^−1^) and 4 concentration of K (0.07, 0.15, 0.3, and 3.0 mmol L^−1^) prepared with deionized water (EC_w_ = 0.05 dS m^−1^). NaCl and K (partially substitute for Na) were added to a basic, modified (no K), ½-strength Hoagland’s solution (Control) containing the same concentrations of macro- and micronutrients and 0.5-3.5 mmol L^−1^ of Na^+^ (Table 2). The solutions were constructed and balanced using the free software ExtractChem V. 2.0 (by Suarez and Taber, Agricultural Water Use Efficiency and Salinity Research Unit in Riverside, or US Salinity Laboratory (USDA-ARS) and available at: https://www.ars.usda.gov/research/software/download/?softwareid=155&modecode=20-36-15-00.

The following salts were used to obtain target salinities in irrigation solution: NaCl, Ca(NO_3_)_2_·4H_2_O, NaNO_3_, KNO_3_, KCl, NaH_2_PO_4_, MgSO_4_·7H_2_O (Table 1). NaFe-EDTA (0.05 mM), H_3_BO_3_ (0.023 mM), MnSO_4_·H_2_O (0.005 mM), ZnSO_4_.7H_2_O (0.0004 mM), CuSO_4_·5H_2_O (0.0003 mM), and (NH_4_)_6_Mo_7_O_24_·4H_2_O (0.0001 mM) were added to every solution to meet plant micronutrient requirements. The concentrations of K^+^ were achieved by substituting varying amounts of KNO_3_ for NaNO_3_ to achieve target K^+^ levels. This resulted in slight decreases in Na^+^ with increasing K+ that are insignificant, relative to the amounts of total Na+ applied, with real values given in Table 2. However, to simplify the presentation of results and discussion, we will refer to the Na doses with the nominal values of 5, 30, 60, 120, and 160 mmol_c_ L^−1^.

### 2.4. Statistical Analyses

Data for each cultivar was used for analyses of variance. Means were compared using Fisher’s LSD test (*p* < 0.05). Statistical analysis was performed using SAEG version 9.1 [45] and the SigmaPlot/SigmaStat version 14.0 (Systat Software, San Jose, CA, USA, www.systatsoftware.com) software. Interactions were analyzed regardless of the significance of the F test. All interactions between Na and K doses were analyzed statistically independently of the significance of the F test. Analysis was also done for individual effects of either Na or K doses for all the parameters related to mineral tissue accumulation and biomass accumulation, except for germination percentage, shoot fresh weight, N, P, Ca, Mg, and S in roots and shoots of both cultivars. These parameters were analyzed taking the F test into consideration, also for individual effects of either NaCl or K doses. Because there was no significant effect of K dose, but there was of Na dose (salinity), the results are presented for salinity inside of each K dose.

## 3. Results

### 3.1. Effect of Combined Salinity and Potassium Doses on Germination and Seedling Establishment

**Figure 1:**
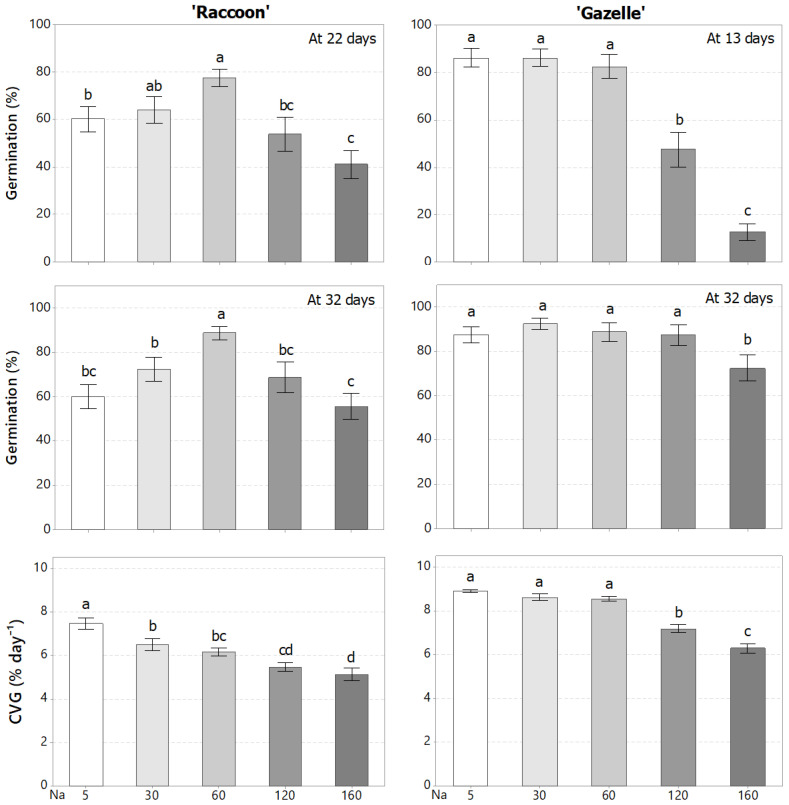
Germination percentage (%) for initial germination count and at 32 days and coefficient of velocity of germination (CVG) for ‘Raccoon’ and ‘Gazelle’ for different doses of NaCl (Na) in mmol_c_ L^−1^. Mean bars with different letters are significantly different by Fisher’s LSD test (*p* < 0.05). Interval bars represent the standard errors of means. Lowercase letters: compare NaCl doses (Na) for germination parameters inside each cultivar. Because there was no effect of K doses, samples were grouped per salinity treatment, with *n* = 16.

There was no effect of K dose or significant interaction between Na and K doses but there was a significant effect of salinity on both germination and shoot fresh weight. During germination and seedling establishment, there was a significant decrease in the percentage of germination (GP) and in the coefficient of velocity of germination (CVG) with increased NaCl-induced salinity for both cultivars, regardless of the K dose. Thus, results were presented in terms of nominal NaCl doses (Figure 1), calling attention to the fact that Cl^−^ concentrations, after the control, increased in parallel with Na^+^ in irrigation waters. At high NaCl doses, GP decreased for both cultivars. However, the GP of ‘Raccoon’ was significantly better than control (5 mmol_c_ L^−1^) at 60 mmol_c_ L^−1^, both at 22 and 32 days after seeding, while ‘Gazelle’ maintained GP up to 60 mmol_c_ L^−1^ at 13 days and up to 120 mmol_c_ L^−1^ at 32 days after germination (Figure 1). The CVG decreased significantly with NaCl dose for both cultivars, with the decrease more pronounced in ‘Raccoon’ than ‘Gazelle’, which maintained its CVG level up to 60 mmol_c_ L^−1^.

**Figure 2:**
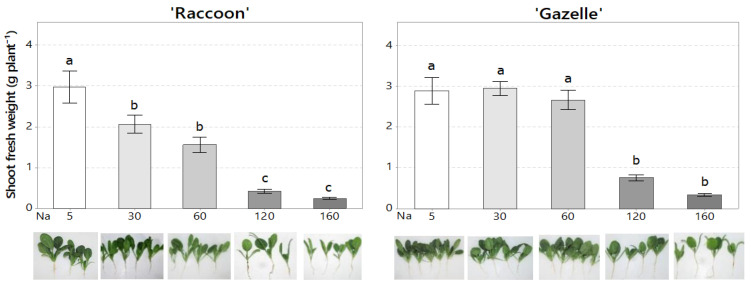
Seedling shoot fresh weight and appearance of ‘Raccoon’ and ‘Gazelle’ seedlings at different NaCl (Na) doses in mmol_c_ L^−1^, regardless of KCl dose (*n* = 16). Mean bars with different letters are significantly different by Fisher’s LSD test (*p* < 0.05). Interval bars show standard errors of means. Plants pictured are from one random pot with 3.0 mmol_c_ L^−1^ K at each Na dose.

Seedling shoot fresh weight decreased for both cultivars as salinity increased (Figure 2), regardless of K dose. However, after 32 days of irrigation with high-salinity water, seedlings had no visual signs of NaCl toxicity (Figure 2).

### 3.2. Effect of Combined Salinity and Potassium Doses on Tissue Na and Cl Accumulation

**Figure 3:**
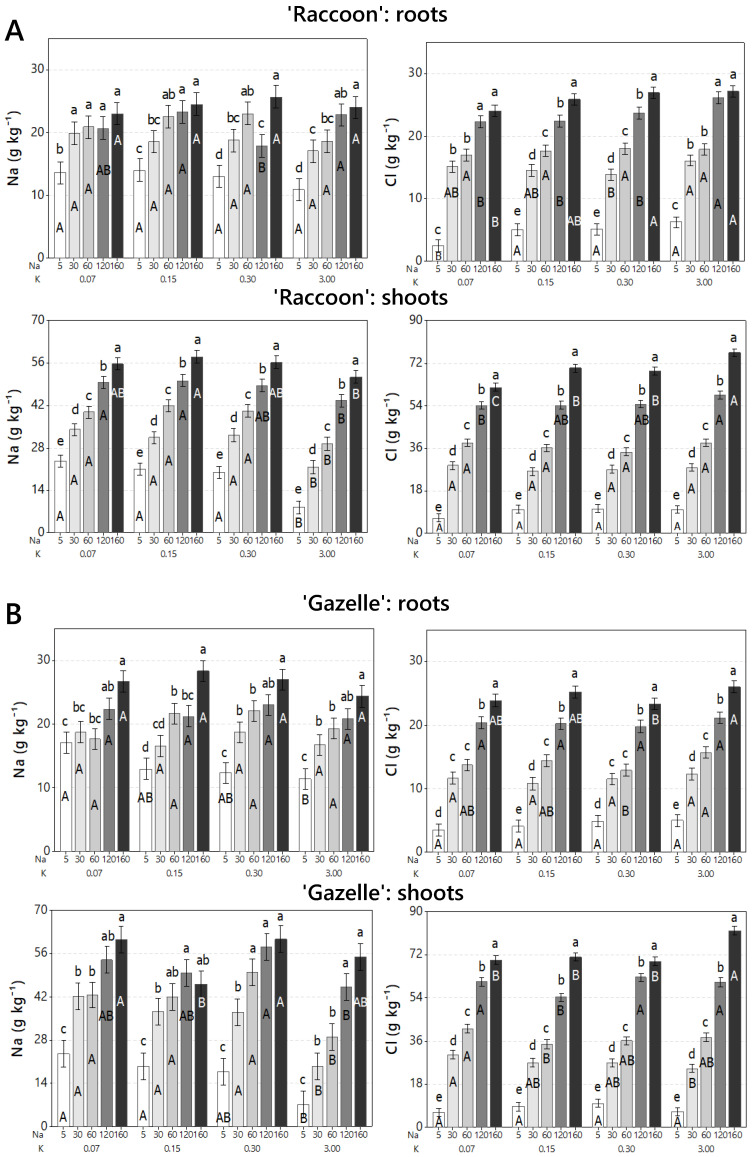
Concentrations of Na and Cl in roots and shoots of ‘Raccoon’ (**A**) and ‘Gazelle’ (**B**) for the respective combinations of NaCl (Na) and KCl (K) doses. Mean bars with different letters are significantly different by Fisher’s LSD test (*p* < 0.05). Interval bars show standard errors of means. Uppercase letters compare K doses within each Na, inside each mineral. Lowercase letters compare NaCl doses (Na), within each K dose, for either Na or Cl.

A second experiment was conducted with seedlings of 6–8 true leaves submitted to the same NaCl concentrations and potassium doses (three deficient and one control) used in the germination study. From here on, all the results are related to this second experiment. The effects of increased NaCl on tissue ionic composition were evaluated through the tissue accumulation of Na and Cl in root and shoot tissues of ‘Raccoon’ and ‘Gazelle’ (Figure 3). In general, both Na and Cl increased with every increase in irrigation-water salinity with both ions accumulating at similar concentrations in roots but with Cl accumulating at higher concentrations in shoots than Na (Figure 3). In roots, from control to the highest salinity treatment, both cultivars showed similar increases of 2-fold for Na (from 13 to 26 g kg^−1^, approximately) and 5.6-fold for Cl (from 4.5 to 25 g kg^−1^, approximately). In shoots, from control to the highest salinity treatment, the accumulation of Na and Cl in ‘Gazelle’ (Figure 3) was similar to that of ‘Raccoon’ and was approximately 3-fold for Na and from 7.6 (‘Raccoon’) to 9.0 (‘Gazelle’) times higher for Cl from control to 160 mmol_c_ L^−1^ NaCl, regardless of K doses (Figure 3).

**Figure 4:**
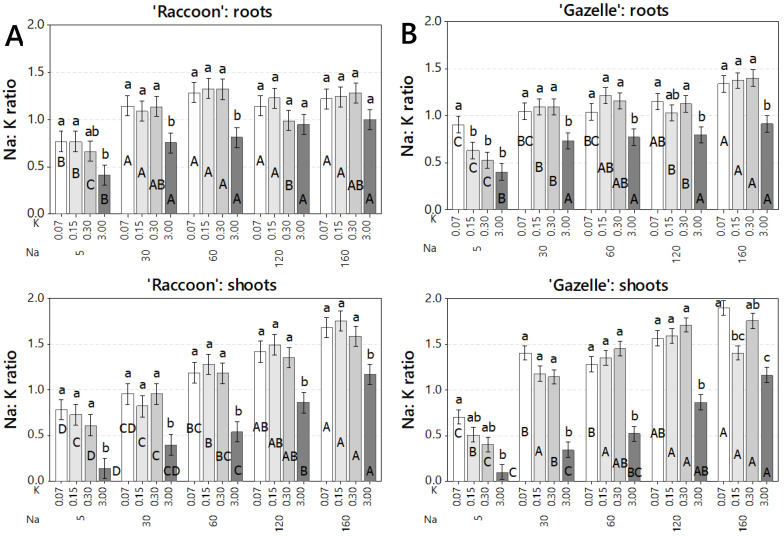
Na:K ratio in roots and shoots of ‘Raccoon’ (**A**) and ‘Gazelle’ (**B**) for the respective combinations of KCl (K) and NaCl (Na) doses (mmol_c_ L^−1^). Mean bars with different letters are significantly different by Fisher’s LSD test (*p* < 0.05). Interval bars show standard errors of means. Uppercase letters compare NaCl (Na) doses, within each K dose, inside organ and cultivar. Lowercase letters compare K doses, within each Na dose, inside organ and cultivar.

For both cultivars the Na:K ratio increased with increased salinity but decreased significantly when K dose increased from deficient (0.07–0.3 mmol_c_ L^−1^) to sufficient (3.0 mmol_c_ L^−1^). Although the decrease lessened at higher NaCl doses, it was still significant for shoots of ‘Raccoon’ and for roots and shoots of ‘Gazelle’ (Figure 4). At control salinity (5 mmol_c_ L^−1^ NaCl) the decrease in Na:K ratio in shoots was seen stepwise, mainly for ‘Gazelle’, despite the low increase in K concentration for deficient doses. At higher salinities, this decrease in Na:K ratio was only observed when K dose increased from deficient to 3.0 mmol_c_ L^−1^ (Figure 4).

### 3.3. Effect of Combined Salinity and Potassium Doses on Root Mineral Composition

**Figure 5:**
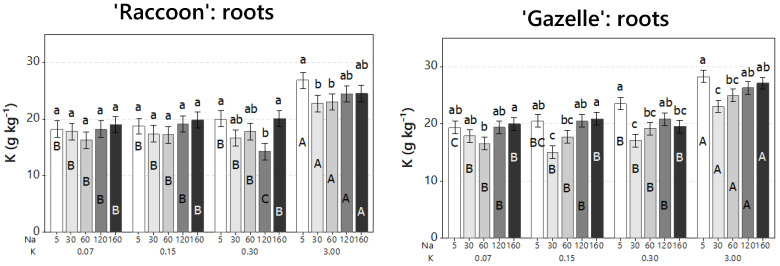
Concentrations of potassium in roots of ‘Raccoon’ (**left**) and ‘Gazelle’ (**right**) for the respective combinations of NaCl (Na) (from 5 to 160 mmol_c_ L^−1^), and KCl (K) doses (from 0.07 to 3.0 mmol_c_ L^−1^). Mean bars with different letters are significantly different by Fisher’s LSD test (*p* < 0.05). Interval bars show standard errors of means. Lowercase letters compare Na doses within each K dose. Uppercase letters compare K doses within each Na dose.

The effects of combined increased NaCl with potassium deficiency was evaluated on root and shoot composition of macronutrients (Table 3) and micronutrients (Supplementary Tables S1 and S2). Despite the significant increase in both Na and Cl, roots maintained their concentrations of K (14.3 to 26.8 g kg^−1^ for ‘Raccoon’ and 15.0 to 28.3 g kg^−1^ for ‘Gazelle’) (Figure 5) and of N (18.6 to 25.2 g kg^−1^ for both cultivars), even when K was applied at deficient doses (Table 3). However, Ca, Mg, and S decreased significantly with salinity by approximately 50%, 32%, and 54%, respectively, in roots of both cultivars (Table 3). Interestingly, P increased significantly with salinity, in each K dose, in roots of ‘Gazelle’. However, the increase was not always significant in roots of ‘Raccoon’ (Table 3).

Regarding root micronutrients, ‘Raccoon’ Fe was the highest, ranging from 820 (highest salinity) to 1350 mg kg^−1^ (control salinity), followed by Mn, which ranged from 254 (highest salinity) to 304 mg kg^−1^ (control salinity), with the remaining micronutrients (B, Cu, and Zn) ranging from 19 to 29.7 mg kg^−1^. Salinity increase led to a significant reduction of Cu (35%), Fe (39%), and Mn (16.5%) but had no effect of the concentrations of B (18–24 mg kg^−1^) or Zn (22 to 29.7 mg kg^−1^) (Table S1). In ‘Gazelle’, Fe and Mn were also the highest micronutrients ranging, respectively, from 975 to 1730 mg kg^−1^ and from 250 to 452 mg kg^−1^, while B ranged from 40–50 mg kg^−1^ and Zn from 19–25 mg kg^−1^. Salinity led to significant reductions in Cu (50%), Fe (44%), Mn (43%), and Zn (24%) (Table S2*)**.***

### 3.4. Effect of Combined Salinity and Potassium Doses on Shoot Mineral Composition

**Figure 6:**
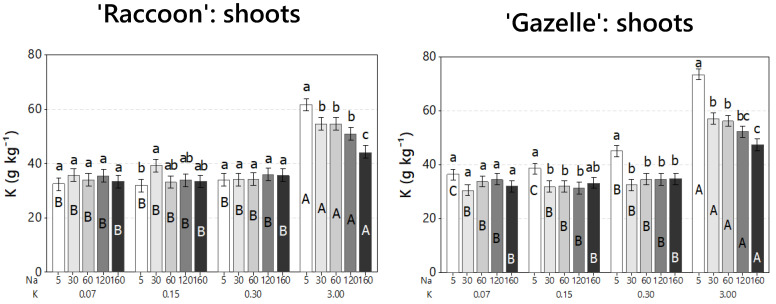
Concentrations of the mineral potassium (K, in g kg^−1^) in shoots for ‘Raccoon’ (**left**) and ‘Gazelle’ (**right**) for the respective combinations of NaCl (Na) (from 5.0 to 160.0 mmol_c_ L^−1^) and KCl (K) doses (from 0.07 to 3.0 mmol_c_ L^−1^). Mean bars with different letters are significantly different by Fisher’s LSD test (*p* < 0.05). Interval bars show standard errors of means. Uppercase letters compare K within each Na dose in shoots of each cultivar. Lowercase letters compare Na doses, within each K dose.

Similar to roots, and regardless of the significant increases in Na and Cl, or K-deficient doses, shoot concentrations of K (Figure 6) and N (Table 3) were maintained and ranged from 31.9 (when K was provided in deficient doses) to 61.6 g kg^−1^ (when K was provided at 3.0 mmol_c_ L^−1^) for ‘Raccoon’ and from 30.5 (deficient K) to 73.5 g kg^−1^ (3.0 mmol_c_ L^−1^ K) for ‘Gazelle’, while N ranged from 43.4 to 49.3 g kg^−1^ for ‘Raccoon’ (Figure 6, left, Table 3) and from 28.5 to 42.0 g kg^−1^ for ‘Gazelle’ (Figure 6, right, Table 3). As observed in roots, P was maintained or slightly increased (at high NaCl doses), while Ca, Mg, and S decreased with salinity in 54%, 31%, and 36%, respectively for ‘Raccoon’ and 53%, 29%, and 29.5%, respectively for ‘Gazelle’ (Table 3). When K was provided at the sufficient dose of 3.0 mmol_c_ L^−1^, there was a significant decrease in shoot K of both cultivars when salinity increased beyond control salinity and mainly from control to the highest salinity, with an average K decrease of 31% for ‘Raccoon’ and 34% for ‘Gazelle’ (Figure 6).

Like root micronutrient concentration, Fe and Mn were the highest micronutrients found in shoots of both cultivars without a trend in their concentration associated with salinity or K dose (Tables S1 and S2).

### 3.5. Effect of Combined Salinity and Potassium Doses on Plant Biomass Accumulation

**Figure 7:**
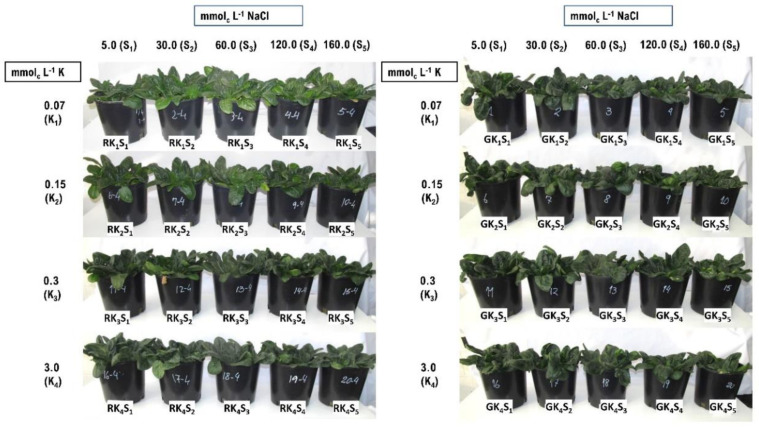
Appearance of spinach plants for ‘Raccoon’ (R, **left**) and’ (G, **right**) seven weeks (50 days) after treatments with the respective combinations of NaCl (5.0 to 160.0 mmol_c_ L^−1^), or salinity treatments S_1_ to S_5_ and potassium, as potassium chloride (KCl) or K (0.07 to 3.0 mmol_c_ L^−1^) doses K_1_ to K_4_. Plants were photographed the same day the experiment was terminated.

**Figure 8:**
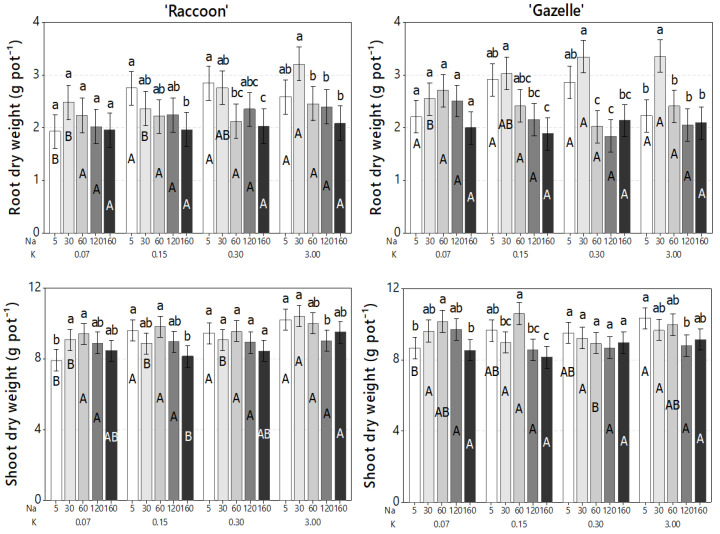
Dry biomass of roots and shoots for ‘Raccoon’ and ‘Gazelle’ for the respective combinations of NaCl (Na) and KCl (K) doses in mmol_c_ L^−1^. Mean bars with different letters are significantly different by Fisher’s LSD test (*p* < 0.05). Interval bars show standard errors of means. Uppercase letters compare K doses within each Na dose, for roots or shoots inside each cultivar. Lowercase letters compare Na doses, within each K dose, for roots or shoots inside each cultivar.

Salinity effects were evaluated on plant vegetative growth and biomass accumulation through plant appearance (Figure 7), RDW, and SDW (Figure 8). Although salinity caused significant reductions in the concentrations of Ca, Mg, and S, these reductions were not enough to cause visual mineral deficiencies, regardless of K and NaCl doses, and allowed the development of healthy-looking spinach plants (Figure 7).

Regarding K dose, ‘Raccoon’ plants had a significant increase in shoot biomass when K increased from 0.07 mmol_c_ L^−1^ to higher K doses. ‘Gazelle’ had a similar response, although significant shoot biomass increase was only significant for the K doses of 0.3 and 3.0 mmol_c_ L^−1^. However, K doses higher than 0.07 mmol_c_ L^−1^ had no effect on biomass of either cultivar when NaCl doses were higher than 30 mmol_c_ L^−1^ (Figure 8). For SDW, at the lowest K dose of 0.07 mmol_c_ L^−1^, plants of both cultivars increased SDW significantly when Na concentration increased from 5.0 to 30 and 60 mmol_c_ L^−1^. The average SDW ranged from 7.9 to 10.4 g pot^−1^ for ‘Raccoon’ and from 8.2 to 10.6 g pot^−1^ for ‘Gazelle’ and, when there was a significant increase or decrease in SDW, the difference was approximately 2.0 g pot^−1^ (Figure 8). Although K doses had little or no effect on root dry weight (RDW) and shoot dry weight (SDW), RDW of ‘Gazelle’ increased significantly at 30 mmol_c_ L^−1^ NaCl when K was sufficient (3.0 mmol_c_ L^−1^). The data showed a similar increase for ‘Raccoon’ at the same NaCl dose (although not significant) as if a small increase in salinity had favored root development (Figure 8).

## 4. Discussion

### 4.1. Effect of Combined Salinity and Potassium Doses on Germination and Seedling Establishment

While there was no effect of K dose on germination, ‘Raccoon’ had its germination improved by salinity while ‘Gazelle’ was more tolerant to salinity than ‘Raccoon’ during germination and seedling establishment. Different researchers cited in the introduction demonstrated that crop germination decreased with increased salinity of irrigation water. The salinity threshold of approximately 120 mmol_c_ L^−1^ NaCl 32 days after germination for ‘Gazelle’ (Figure 1) was higher than that obtained for other cultivars of spinach such as ‘Green Gold’, ‘Larisa’, ‘Mikado’, and ‘Ohio’, in which the GP and relative germination rate were unaffected up to 50 mM (50 mmol_c_ L^−1^) NaCl but decreased significantly at 100 mM NaCl, and further at 200 mM NaCl [20]. Our germination data also indicate that GP improved from the first counting date for each cultivar (22 days for ‘Raccoon’ and 13 days for ‘Gazelle’) to the last counting at 32 days after seeding (Figure 1). Because most of ‘Gazelle’ seeds germinated before ‘Raccoon’s seeds, CVG values for Gazelle were higher than those for ‘Raccoon’. Although not compared statistically, mean GP and CVG for ‘Gazelle’ appeared to be better than those of ‘Raccoon’. Average seedling fresh weight (Figure 2) also indicates that ‘Gazelle’ has a higher salinity tolerance than ‘Raccoon’. Our data suggest that irrigation water salinity below 7.0 dS m^−1^ (60 mmol_c_ L^−1^) would not affect either GP or seedling growth of Gazelle (Figure 2) if irrigation frequency is enough to maintain soil salinity at similar levels of irrigation-water salinity. Although ‘Gazelle’ showed a better recuperation in GP than ‘Raccoon’, seedling fresh weight data (Figure 2) clearly indicated that seedling growth was significantly affected by NaCl doses above 60 mmol_c_ L^−1^ NaCl (7.0 dS m^−1^). It has been previously shown that the lack of response to K fertilization at high levels of NaCl could be explained, at least in part, by the reduction in plant growth caused by NaCl ion toxicity, which limits the absorption of water and nutrients from the soil [46]. Although seedlings did not provide enough material for mineral analysis, they had no visual symptoms of either mineral deficiency or salt toxicity (Figure 2). Furthermore, because of ‘Gazelle’s CVG reflected a faster initial germination than that observed for ‘Raccoon’, seedling biomass observed for ‘Gazelle’ up to 60 mmol_c_ L^−1^ may reflect the fact that ‘Gazelle’s seedlings had more time to adjust to salinity.

### 4.2. Effect of Combined Salinity and Potassium Doses on Tissue Na and Cl Accumulation

The significant increase in both Na and Cl in roots and shoots of both cultivars was expected with increasing NaCl concentrations. The steady and stepwise increase in tissue Cl, which surpassed the increase in tissue Na (Figure 3), indicates that spinach is more efficient in controlling Na than Cl. Increases in both tissue Na and Cl of similar magnitudes have been reported recently when irrigation water contained 90 and 120 mmol_c_ L^−1^ of NaCl [8]. Interestingly, the use of saline water containing 120 and 160 mmol_c_ L^−1^ in this study resulted in Na shoot accumulation of approximately 50–60 g kg^−1^, smaller than the 60–70 g kg^−1^ of shoot Na reported previously [8] for the same cultivars under 90 and 120 mmol_c_ L^−1^ of Na. The same can be said for Cl. Our data indicate that spinach plants have control mechanisms triggered by salinity that allows plants to control salt tissue accumulation once Na and Cl reach a certain level. However, the salinity threshold beyond which the plant will no longer be able to control its tissue accumulation of Na and Cl has not been determined. It is noteworthy to mention that in naturally-saline waters, the concentration of Cl^−^ can be as high to twice as high as the concentration of Na^+^.

The ratio of Na:K is considered an important measure of tolerance of a species or cultivar to salinity. However, in the case of these two spinach cultivars, this ratio only increased with salinity because Na tissue levels increased significantly at every step while K tissue concentrations remained constant (Figure 5 and Figure 6). However, the increase from 0.3 to 3.0 mmol_c_ L^−1^ in K was enough to show that K tissue accumulation was antagonistic to Na tissue accumulation. These results agree with a previous report that concluded that a 20-fold increase in K from 0.25 to 5.0 mmol_c_ L^−1^ decreased Na tissue accumulation [8].

### 4.3. Effect of Combined Salinity and Potassium Doses on Root Mineral Composition

Plants maintained adequate concentrations of N, P, and K in roots, regardless of salinity or K dose. Although Ca, Mg, and S decreased significantly with salinity, regardless of K dose, plants maintained a minimum concentration of these macronutrients needed for growth [8,47]. Root-P increase in both cultivars as salinity increased (Table 3) could have been an attempt for the plant to balance ions using HPO_4_^2−^ to compensate for a constant concentration of NO_3_^−^ in the leaf (interpreted through the constant tissue levels of mineral N), and in response to a steady increase of Na^+^ in saline irrigation waters, as suggested by others [48]. Similar results were reported for ‘Raccoon’ and ‘Gazelle’ in previous salinity studies either when K was provided at sufficient doses [6] or when mineral composition was compared under salinity with K provided at both sufficient and deficient doses [8].

Regarding root micronutrients, Fe and Mn were the highest among other micronutrients tested. Although salinity significantly reduced Fe, Cu, and Mn (and Zn in ‘Gazelle’), these micronutrients still were present at sufficient concentrations for root development. In a study evaluating the effect of combined salinity with N doses in spinach, the authors reported a significant decrease in Fe with increased salinity, in agreement with our results but, contrary to our results, they reported small, but significant, increases in Zn and Cu as salinity increased [49], which could be a cultivar-specific response. Although other micronutrients were present at similar concentration in roots and shoots, Mn and Fe were found in roots in concentrations 6-fold or higher and 2.5-fold or higher in roots than in shoots, respectively. The data on mineral composition of spinach roots suggest that, instead of being discarded, they should be used as a rich source of Fe, Mn, and of macronutrients for human and animal diets.

### 4.4. Effect of Combined Salinity and Potassium Doses on Shoot Mineral Composition

Our shoot macronutrient data for both ‘Raccoon’ and Gazelle clearly established that concentrations of N, P, and K were maintained in shoots across salinity levels (Table 3; Figure 6). The increase in shoot P concentration, mainly observed in ‘Gazelle’ may have been an attempt for the plant to balance HPO_4_^2−^ ions under an increased concentration of Na^+^, as previously discussed under root mineral composition, and as suggested by others [48]. Similar results on the maintenance of N concentrations and increased P concentrations in spinach shoots, under similar salinity levels, were reported by others [49]. It has been established earlier that differences in salt tolerance of three brassica species could be related to K^+^ retention in roots [50]. The high salt tolerance of ‘Raccoon’ and ‘Gazelle’ may be attributed to their ability to retain K^+^ in both roots and shoots, regardless salinity increase in irrigation waters or the low availability of K in irrigation waters. This K homeostasis against its availability in soil or water was discussed at the molecular level elsewhere [8].

Interestingly, shoot micronutrients, in general, showed no significant differences in either cultivar as salinity increased. The concentrations of Fe and Mn were also the highest, as they were in roots, but at much lower magnitudes (Tables S1 and S2).

Homeostasis of N, P, and K was maintained in both spinach cultivars, regardless of the significant increase in Na and Cl and regardless the fact that three of the K doses were from 10 to 43 times lower than what was considered sufficient for spinach growth in previous works [6,40]. The concentrations of shoot Ca, Mg, and S, although with significant decreases, were still maintained at levels high enough for plant growth and shoot biomass accumulation, similar to our previous results comparing two doses of K (0.25 and 5.0 mmol_c_ L^−1^), also under elevated irrigation-water salinity [8].

### 4.5. Effect of Combined Salinity and Potassium Doses on Plant Biomass Accumulation

A similar increase in shoot biomass under low to mild salinity, as the one observed in this study, was reported for ‘Raccoon’ when irrigation salinity significantly increased total dry matter (roots + shoots) at 30 and 60 mmol_c_ L^−1^ [8]. The SDW increase observed with 30 and 60 mmol_c_ L^−1^ at the lowest K dose suggests that plants of both cultivars benefited from NaCl when K was deficient (Figure 8). Although salinity increase caused a decrease in RDW at the K doses of 0.15 and 0.30 mmol_c_ L^−1^, SDW remained mostly unchanged, regardless of K dose and salinity levels. However, a small, but significant, decrease in shoot biomass of both cultivars was observed at 0.15 mmol_c_ L^−1^ K between control salinity and 160 mmol_c_ L^−1^ NaCl. Thus, in general, neither K nor NaCl dose had a great impact on SDW of either cultivar.

Previous results with the same cultivars grown in soil under lower NaCl concentrations (from 5.0 to 120 mmol_c_ L^−1^) showed a significant decrease in dry shoot biomass for ‘Raccoon’ after 30 mmol_c_ L^−1^ when K was 5.0 mmol_c_ L^−1^, while ‘Raccoon’ significantly increased SDW with 30–60 mmol_c_ L^−1^ NaCl when K was 0.25 mmol_c_ L^−1^ [8]. This significant increase in shoot biomass with 30–60 mmol_c_ L^−1^ was also observed for both ‘Raccoon’ and ‘Gazelle’ when K dose was 0.07 mmol_c_ L^−1^ in this experiment (Figure 8), 3.6-fold less than the deficient dose used by Ferreira and collaborators [8]. Plants in that experiment were cultivated in 1:1 (loamy sandy soil:sand) and submitted to salinity when they had 6–8 true leaves, as in this experiment. Because of the use of loamy sand soil and a leaching fraction of 0.25 (25% leaching) used by those authors, plants irrigated with 90 and 120 mmol_c_ L^−1^ were exposed to soil salinities (EC_e_) of 8.9 to 10.4 dS m^−1^, respectively, at the end of the experiment. These soil–paste salinities correspond to irrigation-water salinities (EC_iw_) of 19.6 and 22.9 dS m^−1^, respectively, thus explaining the significant decrease in shoot biomass at these two salinities. In this study, plants were cultivated in an all-sand medium to reduce K to deficient levels for seven weeks under the salinity treatments, and a leaching fraction of 0.30 was applied. Thus, the low effect of salinity on SDW, even at 160 mmol_c_ L^−1^ (EC_iw_ = 17 dS m^−1^), can be partly explained by the fact that the sandy medium allowed a more efficient leaching of NaCl, preventing salinity build-up in the pot, and partly because spinach plants may have adapted to NaCl during the seven weeks (50 days) of cultivation, while in a previous experiment with ‘Raccoon’, plants were only allowed to grow under salinity for only 28 days [6].

Our results clearly indicate that K requirements of spinach are much lower relative to the ones previously recommended for the crop (63 to 138 kg ha^−1^ or 1.6 to 3.54 mmol_c_ L^−1^) [41]. Despite the fact that the lowest K level of 0.07 mmol_c_ L^−1^ used (equivalent to 2.73 kg of K ha^−1^) was from 23 to 50 times lower than recommended for spinach fertilization [41], plants maintained N, P, and K homeostasis in both roots and shoot, thus sustaining growth. Our results on K requirements confirm previously-reported data with spinach, in which plant biomass was similar under both 0.25 and 5.0 mmol_c_ L^−1^ of K combined to a soil salinity of EC_e_ = 6.0 dS m^−1^ [8], equivalent to the EC_iw_ of 13.2 dS m^−1^ in this study. However, our results disagree with a previous study that reported that spinach plants benefitted from extra K when salinity increased from 50 to 250 mM NaCl [51]. In a salinity experiment with ‘Raccoon’ using sufficient K doses of 3, 5, and 7 mmol_c_ L^−1^, plants grew well up to 80 mmol_c_ L^−1^ NaCl (EC_iw_ = 9.3–9.8 dS m^−1^), regardless of the K doses and had no significant decrease in plant dry weight [6]. A study where spinach was cultivated in a loamy sand soil testing K doses of 0, 63, 85, 127, and 148 kg ha^−1^, without salinity, reported that spinach shoot dry weight showed no difference among all the K doses tested [39]. However, those authors did not consider that the loamy soil used could have enough K for spinach to grow without any additional K needed in the control dose of 0.0 kg ha^−1^. In our experiment, spinach shoot biomass was not generally affected by salinity up to 160 mmol_c_ L^−1^, despite significant shoot reductions in Ca, Mg, and S, and regardless of the very low doses of K provided. Thus, spinach plants behaved unlike what was expected for glycophytic plants irrigated with water electrical conductivity (EC_iw_) up to 17 dS m^−1^ (160 mmol_c_ L^−1^ NaCl) combined with deficient K in the growth medium.

Our results show clear differences in shoot biomass between seedlings and older spinach plants in response to the same water-salinity treatments. Plants exposed to salinity at the 6–8 true-leaf stage and allowed to grow for seven weeks into adult plants were more salt-tolerant than seedlings. Eggplant varieties subjected to increasing salinity during germination and seedling stages also responded differently depending on plant growth stage, with salinity tolerance of all cultivars increasing at the later growth stages [36]. Spinach plants, even under very low levels of K maintained optimal plant growth, suggesting that there are genetic mechanisms that are triggered under either low K, or both low K and high salinity, that are responsible for K and N, and maybe P, homeostasis in the plant as previously discussed [8,52,53]. Our results suggest that spinach plants can tolerate moderate to high salinity in sandy soils, and when applied a leaching fraction of approximately 0.30, having the ability to adjust to some degree of salinity as plants grow older. Thus, saline waters would be better suited for the production of “freezer spinach” (older plants) than of “baby spinach” as the former is harvested at a later developmental stage with mineral nutrient concentrations that can be twice that of baby spinach [41]. However, these results obtained in sand culture must be interpreted carefully and cannot be directly extrapolated to field cultivation under different leaching regimes before experimenting with the target soil to be used for spinach cultivation. Results clearly suggest that the salinity threshold of spinach, even under deficient levels of K in the soil solution, is higher than the previously reported EC_e_ of 2.0 dS m^−1^ (estimated EC_iw_ = 4.4 dS m^−1^) [54,55,56]. Based on the EC_iw_ of 17 dS m^−1^ tested here, we can estimate an EC_e_ = 7.7 dS m^−1^ for ‘Raccoon’ and Gazelle, or 3.85-fold higher than the EC_e_ provided for spinach (no cultivar mentioned) by the previous authors.

## 5. Concluding Remarks

In general, spinach did not respond to K doses either during germination or vegetative growth and there was no interaction between salinity and K. However, salinity decreased germination parameters of Gazelle at the highest NaCl dose, and drastically reduced seedling biomass at every increase in NaCl for ‘Raccoon’ but only at 120 and 160 mmol_c_ L^−1^ for Gazelle, indicating that Gazelle was more tolerant to salinity than ‘Raccoon’ during germination and seedling establishment, the most salt-susceptible stage. Interestingly, germination % of ‘Raccoon’ improved significantly at 60 mmol_c_ L^−1^ NaCl, suggesting that moderate salinity can improve the germination of this cultivar. Our results also indicate that the responses of spinach to salinity depends on the plant growth stage and on the cultivar. At the later growth stage, ‘Raccoon’ and Gazelle were both tolerant to irrigation waters of moderate to high salinity.

Mature plants of both cultivars accumulated large concentrations of Na and Cl in both roots and shoots, and these ions could have contributed to osmotic balance to maintain cell turgor without causing symptoms of ion toxicity. Both cultivars maintained their macro and micronutrient at adequate, or close to adequate, tissue levels for growth. Even under severe K deficiency (0.07 mmol_c_ L^−1^, equivalent to a range of 23- to 50-fold less K than recommended for the crop), adult plants maintained K concentrations over the minimum required (20 g kg^−1^ SDW) for plant growth. Therefore, mature plants are more salt tolerant than young plants and saline waters would be better adapted to the production of “freezer spinach” (vegetatively mature plants) instead of “baby spinach” (young plants).

The increase in K from deficient to sufficient reduced the Na:K ratio in the shoots, regardless of the level of salinity but the beneficial effects of K on RDW and SDW were observed only at lowest level of salinity (5 mmol_c_ L^−1^ NaCl). This demonstrates that the improvement in the nutritional status of the plant does not overcome the osmotic effects associated with the increase in the total concentration of salts in the root zone.

Plants from both cultivars seemed to benefit from salinity at the lowest K dose of 0.07 mmol_c_ L^−1^ with shoot biomass increasing significantly at 60 mmol_c_ L^−1^ of NaCl. Although not significant, a similar trend for increased root biomass was also observed for both cultivars. Although we assume that this salinity benefit was only caused by Na^+^ (ions), as an osmoticum to maintain cell turgor but not as a substitute of K^+^ in its physiological function, salinity treatments above control levels had similar concentrations of Cl^−^. Regarding mineral tissue accumulation, Cl tissue accumulation was as substantial as that of Na. Interestingly, Cl is recognized to be essential to plant growth only at micronutrient levels (mg kg^−1^), while Na is a needed nutrient for the growth of halophytes, but not of glycophytes, such as spinach. The spinach cultivars studied here have demonstrated to be salt tolerant and our results for Na:K ratio confirmed the results of a previous study with the same cultivars that reported that sufficient K decreases Na tissue accumulation.

Of course, these results cannot be directly extrapolated to field cultivation of spinach in general because salinity tolerance depends on the cultivar, type of soil, season, and irrigation frequency applied. What is clear is that K fertilization can be significantly decreased to increase farmer profits. These results also raise the question: Is K the only mineral that can be drastically reduced in spinach cultivation or would similar shoot biomass be achieved with N reduction? At a time that more food is needed to feed a growing world population and freshwater becomes scarcer and more expensive, or is unavailable, for irrigation in arid and semiarid areas of the planet, we must revisit mineral needs for crops cultivated under saline irrigation as a way to maintain food production, increase farmer’s profits, while decreasing agriculture footprint and slowing down the salinization of our soils and water resources.

Spinach seems to be a salt-tolerant glycophyte in a family (Amaranthaceae) that has halophytes such as *Salicornia* spp., *Sarcocornia* spp., *Suaeda* spp., *Chenopodium* spp., and *Atriplex* spp., which have all adapted to saline and arid environments. The fact that germination percent improved with time and that plants showed no significant decrease in biomass at the adult stage, regardless of K dose and after 50 days of irrigation with moderate to high-salinity waters lead us to conclude that spinach can be successfully cultivated with saline recycled waters in arid and semiarid regions and requires much less K fertilization than previously recommended.

## Figures and Tables

**Table 1:** Average (minimum/maximum) relative humidity (ARH), temperature, and light intensity as photosynthetic photon flux density (PPFD) per month in a greenhouse used for the germination and growth of spinach cv. Raccoon and Gazelle. Fall and winter in California were from 23 September–21 December 2018 and from 22 December 2018–19 March 2019.

Date	ARH (%)	Temp (°C)	PPFD * (µmol m^−2^ s^−1^)	Day Length ** (h:min:s)
October 19/2018 (min/max)	5.0/66.0	22.2/31.8	433.0/624.4	11:12:36
November/2018 (min/max)	3.0/73.4	19.8/30.1	224.4/517.6	10:04:37
December/2018 (min/max)	10.0/71.0	15.6/28.0	222.8/423.0	09:56:06
January/2019 (min/max)	8.0/69.0	14.6/27.8	200.0/458.6	10:31:53
February/2019 (min/max)	18.0/61.3	13.1/31.1	427.0/634.4	11:25:28
March 11/2019 (min/max)	30.0/66.0	17.0/30.7	351.6/771.2	11:48:34

* PPFD was estimated for 8.5–9.0 h day^−1^ (from 7:00 a.m. to 5:00 p.m.) with approximately half the hours averaged for minimum and half for maximum PPFD. Sensor was from Apogee Instruments, Inc. (Logan, UT, USA). ** Daylength data are given from 19 October 2018 to 11 March 2019, and for the last day of each month, except for October and March (daylength for 1st and last day of experiment only). Daylength data source: https://www.timeanddate.com/sun/usa/riverside.

**Table 2:** Cation and anion concentrations of saline solutions used to irrigate seeds (germination experiment) for 32 days (up to 4–6 true-leaf stage) and young plants bearing 6–8 true leaves for seven weeks (growth experiment).

Treatment	K^+^	Na^+^	Cl^−^	H_2_PO_4_^−^	Ca^2+^	Mg^2+^	SO_4_^2−^	NO_3_^−^	TEC_iw_	MEC_iw_	pH
	(mmol_c_ L^−1^)								(dS m^−1^)		
T1	0.07	3.5	0.07	0.5	5.0	2.0	2.0	8.0	1.0	1.3	6.14
T2	0.07	30.5	27.07	0.5	5.0	2.0	2.0	8.0	4.0	4.1	6.05
T3	0.07	60.5	57.07	0.5	5.0	2.0	2.0	8.0	7.2	7.2	6.15
T4	0.07	120.5	117.07	0.5	5.0	2.0	2.0	8.0	13.2	12.7	6.07
T5	0.07	160.5	157.07	0.5	5.0	2.0	2.0	8.0	17.1	16.8	6.09
T6	0.15	3.5	0.15	0.5	5.0	2.0	2.0	8.0	1.1	1.2	6.22
T7	0.15	30.5	27.15	0.5	5.0	2.0	2.0	8.0	4.0	4.0	6.17
T8	0.15	60.5	57.15	0.5	5.0	2.0	2.0	8.0	7.2	7.2	6.15
T9	0.15	120.5	117.15	0.5	5.0	2.0	2.0	8.0	13.2	13.2	6.18
T10	0.15	160.5	157.15	0.5	5.0	2.0	2.0	8.0	17.1	16.8	6.12
T11	0.3	3.5	0.30	0.5	5.0	2.0	2.0	8.0	1.1	1.3	6.19
T12	0.3	30.5	27.30	0.5	5.0	2.0	2.0	8.0	4.1	4.1	6.17
T13	0.3	60.5	57.30	0.5	5.0	2.0	2.0	8.0	7.2	7.4	6.21
T14	0.3	120.5	117.30	0.5	5.0	2.0	2.0	8.0	13.2	13.2	6.16
T15	0.3	160.5	157.30	0.5	5.0	2.0	2.0	8.0	17.1	16.9	6.09
T16	3.0	0.5	0.00	0.5	5.0	2.0	2.0	8.0	1.1	1.2	6.25
T17	3.0	27.5	27.00	0.5	5.0	2.0	2.0	8.0	4.1	4.8	6.17
T18	3.0	57.5	57.00	0.5	5.0	2.0	2.0	8.0	7.3	7.2	6.18
T19	3.0	117.5	117.00	0.5	5.0	2.0	2.0	8.0	13.2	13.2	6.18
T20	3.0	157.5	157.00	0.5	5.0	2.0	2.0	8.0	17.1	16.7	6.13

TEC_iw_: Target electric conductivity of treatment irrigation water (EC_iw_), MEC_iw_: Measured EC_iw_ in treatment irrigation waters.

**Table 3:** Mean concentrations of macronutrients in roots and shoots of ‘Raccoon’ and ‘Gazelle’ for the respective doses of NaCl (5.00 to 160.00 mmol_c_ L^−1^) pooled for all KCl doses due to lack of K effect (*n* = 16).

Nutrient	*Roots*					*Shoots*				
	NaCl Doses (mmol_c_ L^−1^)									
	5.0	30.0	60.0	120.0	160.0	5.0	30.0	60.0	120.0	160.0
	**‘Raccoon’**									
**N** (%)	2.42A	2.10B	2.19B	2.17B	2.35A	4.82A	4.50C	4.61BC	4.65ABC	4.70AB
**P** (g kg^−1^)	4.96B	4.86B	5.41AB	5.38B	5.95A	4.88AB	4.74B	4.88B	4.92AB	5.17A
**Ca** (g kg^−1^)	9.01A	7.33B	6.27C	4.28D	4.49D	15.21A	10.09B	7.56C	7.11C	7.03C
**Mg** (g kg^−1^)	11.50A	9.74B	8.65C	6.73D	7.59D	10.95A	9.78B	8.84C	7.88D	7.55D
**S** (g kg^−1^)	5.36A	3.78B	3.49B	2.11C	2.39C	4.98A	4.32B	4.06C	3.44D	3.24D
	**‘Gazelle’**									
**N** (%)	2.27AB	2.02D	2.09CD	2.23BC	2.42A	3.94A	3.60A	3.47A	3.61A	3.97A
**P** (g kg^−1^)	5.32CD	5.08D	5.78BC	6.17AB	6.62A	4.57BC	4.31C	4.58B	4.60B	5.04A
**Ca** (g kg^−1^)	8.52A	6.68B	5.89C	4.52D	4.20D	10.90A	7.64B	6.30C	5.45D	5.04D
**Mg** (g kg^−1^)	10.62A	8.85B	8.29B	7.48C	7.57C	9.71A	8.32B	8.10B	7.32C	6.94C
**S** (g kg^−1^)	4.82A	3.59B	3.33B	2.60C	2.31C	4.40A	4.00B	3.79B	3.29C	3.11C

Means with different letters are significantly different by Fisher’s LSD test (*p* < 0.05). Uppercase letters: comparisons between NaCl doses, within each cultivar.

## Supplementary Materials

The following are available online at https://www.mdpi.com/2223-7747/9/12/1739/s1, Table S1: Concentrations of micronutrients in roots and shoots of ‘Raccoon’ for the respective combinations of NaCl (5.0 to 160.0 mmol_c_ L^−1^) and K doses (0.07 to 3.0 mmol_c_ L^−1^), Table S2: Concentrations of micronutrients in roots and shoots of ‘Gazelle’ for the respective combinations of NaCl (5.0 to 160.0 mmol_c_ L^−1^) and K doses (0.07 to 3.0 mmol_c_ L^−1^).
