# Supplementary material for: Germination and Growth of Spinach under Potassium Deficiency and Irrigation with High-Salinity Water
Source: Plants (Basel). 2020 Dec 9;9(12):1739. doi: 10.3390/plants9121739 (PMC7763614; doi:10.3390/plants9121739)
Supplement: Supplementary file 1 [file plants-09-01739-s001.pdf]

Supplementary data

Supplementary Table 1. Concentrations of micronutrients in roots and shoots of 'Raccoon' for the respective combinations of NaCl (5.0 to 160.0 $mmol_c$ L^{-1}) and K doses (0.07 to 3.0 mmolc L^{-1}).

	Root					***Shoot***				
	**NaCl doses** ($mmol_c$ L^{-1})									
**K doses** ($mmol_c$ L^{-1})	**5.0**	**30.0**	**60.0**	**120.0**	**160.0**	**5.0**	**30.0**	**60.0**	**120.0**	**160.0**
	**B** (mg kg^{-1})									
**0.07**	33.15aA	16.25aB	18.92aB	14.64aB	19.97aB	40.63aA	26.59aB	31.41aAB	25.05aB	25.32aB
**0.15**	22.44bA	24.87aA	21.58aA	17.40aA	19.12aA	37.51aA	28.07aAB	24.55abB	23.90aB	29.00aAB
**0.30**	20.98bA	18.10aAB	20.80aA	9.66aB	17.57aAB	39.51aA	24.05aB	18.52bB	20.80aB	19.65aB
**3.00**	21.78bA	19.05aA	16.49aA	16.64aA	16.44aA	31.02aA	31.05aA	20.54abAB	19.59aB	22.53aAB
	**Cu** (mg kg^{-1})									
**0.07**	21.73abA	13.62bB	15.99aB	13.97aB	16.03aB	6.35bA	6.13aA	8.23aA	9.23aA	8.86aA
**0.15**	22.38aA	18.26aAB	16.15aBC	12.03aC	13.76aC	7.14bA	6.51aA	7.48aA	6.19aA	7.27aA
**0.30**	21.02abA	17.19abAB	14.16aBC	7.54bD	11.73aCD	8.31abA	7.10aA	5.27aA	6.60aA	7.88aA
**3.00**	17.64bA	15.35abAB	14.66aAB	11.48abB	12.66aB	12.78aA	5.89aB	5.98aB	5.91aB	7.92aB
	**Fe** (mg kg^{-1})									
**0.07**	1469.03aA	1395.79aA	1242.51aAB	569.12aC	673.90aBC	463.62aA	362.84aA	394.83bA	473.61aA	292.24aA
**0.15**	1556.31aA	1027.27abAB	900.82aB	642.22aB	1002.10aAB	430.43aB	237.00aB	791.15aA	440.40aB	298.49aB
**0.30**	1302.78aA	970.12abAB	866.72aAB	585.86aB	887.12aAB	502.15aA	431.21aA	245.21bA	526.60aA	371.19aA
**3.00**	1089.68aA	766.74bA	860.48aA	800.54aA	715.06aA	379.68aA	417.99aA	351.15bA	218.31aA	212.92aA
	**Mn** (mg kg^{-1})									
**0.07**	257.73abC	500.85aA	374.06aB	343.37aC	244.43aC	111.04aC	160.82abA	144.95bAB	135.66aB	127.02aBC
**0.15**	362.63aAB	420.99aA	445.18aA	291.96aBC	246.73aC	130.16aB	163.88aA	172.40aA	118.31aBC	105.60aC
**0.30**	248.49bBC	483.11aA	344.56aB	216.89aC	314.79aBC	119.68aB	169.47aA	117.28cB	113.25aB	108.89aB
**3.00**	347.26abA	414.31aA	359.31aA	220.16aB	210.86aB	132.11aAB	137.17bAB	152.17abA	123.49aBC	104.88aC
	**Zn** (mg kg^{-1})									
**0.07**	31.02aAB	26.37aBC	24.55aC	21.87abC	34.13aA	31.43bA	29.49aA	31.75aA	33.85aA	41.97aA
**0.15**	32.51aA	22.46aC	23.47aBC	21.15abC	28.67abAB	44.36aA	29.09aB	29.07aB	26.64aB	38.88abAB
**0.30**	32.07aA	21.55aBC	23.17aB	15.94bC	21.68cBC	44.11aA	29.33aB	25.80aB	26.68aB	27.86bB
**3.00**	23.33bA	21.05aA	24.21aA	23.70aA	24.82bcA	28.48bA	27.79aA	27.50aA	29.11aA	38.62abA

Means with different letters are significantly different from each other by Fisher's LSD test ($p < 0.05$). Lowercase: comparisons between K doses, within each Na dose and micronutrient. Uppercase: comparisons between Na doses, within each K dose inside each micronutrient.

Supplementary Table 2. Concentrations of micronutrients in roots and shoots of 'Gazelle' for the respective combinations of NaCl (5.0 to 160.0 $mmol_c$ L^{-1}) and K doses (0.07 to 3.0 mmolc L^{-1}).

**K doses** ($mmol_c$ L^{-1})	***Root***					***Shoot***				
	**NaCl doses** ($mmol_c$ L^{-1})									
	**5.0**	**30.0**	**60.0**	**120.0**	**160.0**	**5.0**	**30.0**	**60.0**	**120.0**	**160.0**
	**B** (mg kg^{-1})									
**0.07**	53.84aA	42.55aB	44.08aAB	38.78aB	40.79aB	50.95aA	50.09aA	51.12aA	47.49aA	44.74aA
**0.15**	45.06aA	37.40aA	39.98aA	37.80aA	41.54aA	54.26aA	51.86aA	46.50aA	49.25aA	53.68aA
**0.30**	51.60aA	46.30aAB	47.83aAB	41.03aB	40.31aB	55.67aA	56.08aA	57.37aA	55.39aA	50.77aA
**3.00**	47.74aA	42.66aA	43.00aA	42.58aA	40.48aA	62.71aA	53.08aAB	49.44aB	49.07aB	48.49aB
	**Cu** (mg kg^{-1})									
**0.07**	17.18abA	10.99aABC	13.48aAB	8.83aBC	7.06aC	8.66aA	8.37aA	8.96bA	9.30aA	8.10aA
**0.15**	12.81bA	8.64aA	11.24aA	8.78aA	8.51aA	8.73aB	8.99aB	18.50aA	8.01aB	8.61aB
**0.30**	12.32bA	10.47aA	9.96aA	8.20aA	8.78aA	6.56aA	7.06aA	7.63bA	7.44aA	7.71aA
**3.00**	19.33aA	10.28aB	9.29aB	9.16aB	7.65aB	6.47aA	8.53aA	6.68bA	6.78aA	7.70aA
	**Fe** (mg kg^{-1})									
**0.07**	1601.20aA	1134.26aB	1328.93aAB	1014.99abB	1068.39aB	260.68aB	289.08aB	253.99bB	472.72aA	281.82aB
**0.15**	1943.66aA	1213.78aB	1100.62aBC	687.97bC	960.81aBC	219.81aB	187.92aB	452.06aA	269.09bB	250.36aB
**0.30**	1683.09aA	1141.16aB	930.07aB	820.65abB	837.95aB	226.82aA	213.51aA	290.49abA	208.49bA	187.14aA
**3.00**	1676.00aA	1033.80aB	1112.79aB	1171.67aB	1033.06aB	203.42aA	192.45aA	204.16bA	331.50abA	214.37aA
	**Mn** (mg kg^{-1})									
**0.07**	372.31aA	347.27aA	383.64aA	275.97aA	312.90aA	102.04bB	122.07aAB	142.96aA	111.37aAB	112.81aAB
**0.15**	487.25aA	390.48aAB	334.35aAB	256.95aB	254.83aB	108.89abAB	134.11aA	125.19aAB	93.70aB	94.53aB
**0.30**	524.83aA	497.30aA	382.45aAB	278.63aB	279.71aB	126.20abAB	133.53aA	109.03aAB	104.38aAB	95.00aB
**3.00**	414.49aAB	416.68aA	290.92aABC	260.34aBC	184.87aC	139.37aA	140.25aA	117.00aAB	92.38aB	81.87aB
	**Zn** (mg kg^{-1})									
**0.07**	23.33aA	19.56aA	19.18aA	20.64bA	19.84aA	25.88aA	24.02aA	26.46aA	27.75aA	25.06aA
**0.15**	25.40aA	15.80aA	18.55aA	19.62bA	19.90aA	25.37aAB	25.89aAB	31.95aA	24.69aB	25.62aAB
**0.30**	25.34aB	18.75aB	20.25aB	36.68aA	18.87aB	25.65aA	24.39aA	26.58aA	23.07aA	23.46aA
**3.00**	27.50aA	21.20aA	24.33aA	22.83bA	18.72aA	23.63aA	27.78aA	25.40aA	24.60aA	25.49aA

Means with different letters are significantly different from each other by Fisher's LSD test ($p < 0.05$). Lowercase: comparisons between K doses, within each Na dose and micronutrient. Uppercase: comparisons between Na doses, within each K dose inside each micronutrient.
